# Retraction Note: Contribution of anthocyanin pathways to fruit flesh coloration in pitayas

**DOI:** 10.1186/s12870-021-03005-6

**Published:** 2021-05-20

**Authors:** Ruiyi Fan, Qingming Sun, Jiwu Zeng, Xinxin Zhang

**Affiliations:** Institute of Fruit Tree Research, Guangdong Academy of Agricultural Sciences; Key Laboratory of South Subtropical Fruit Biology and Genetic Resource Utilization (MOA); Guangdong Province Key Laboratory of Tropical and Subtropical Fruit Tree Research, Guangzhou, 510640 China

**Retraction Note: BMC Plant Biol 20, 361 (2020)**

**https://doi.org/10.1186/s12870-020-02566-2**

The authors have retracted this article. Following publication concerns were raised with the reproducibility of the results. The authors have stated that the RNA-Seq company made a mistake when performing the transcriptome assembly using the mixed samples. In this article the pitaya with red flesh or white flesh belongs to the same genus but different species, and the transcriptome analysis should be separately analyzed. That means that the data on the transcriptome analysis and gene expression in this article are not correct. The authors intend to resubmit a revised manuscript which will undergo peer review.

All authors agree to this retraction.

